# Self-Assembled 1-Octadecanethiol Membrane on Pd/ZnO for a Selective Room Temperature Flexible Hydrogen Sensor

**DOI:** 10.3390/mi13010026

**Published:** 2021-12-26

**Authors:** Pawan Pathak, Hyoung Jin Cho

**Affiliations:** Department of Mechanical and Aerospace Engineering, University of Central Florida, Orlando, FL 32816, USA

**Keywords:** ZnO, palladium nanoparticles, flexible sensors, hydrogen sensors, 1-octadecanethiol

## Abstract

A layer of self-assembled 1-octadecanethiol was used to fabricate a palladium (Pd)/zinc oxide (ZnO) nanoparticle-based flexible hydrogen sensor with enhanced response and high selectivity at room temperature. A palladium film was first deposited using DC sputtering technique and later annealed to form palladium nanoparticles. The formation of uniform, surfactant-free palladium nanoparticles contributed to improved sensor response towards hydrogen gas at room temperature. The obtained sensor response was higher than for previously reported room temperature Pd/ZnO sensors. Furthermore, the use of the polymer membrane suppressed the sensor’s response to methane, moisture, ethanol, and acetone, resulting in the selective detection of hydrogen in the presence of the common interfering species. This study shows a viable low-cost fabrication pathway for highly selective room temperature flexible hydrogen sensors for hydrogen-powered vehicles and other clean energy applications.

## 1. Introduction

Hydrogen (H_2_) gas has been regarded as one of the promising next-generation clean-energy sources because it exhibits a threefold energy density compared to that of gasoline and its combustion reaction produces water (H_2_O) as the only byproduct [1,2]. Therefore, with the foreseeable depletion of fossil fuels, the field of hydrogen-powered fuel cell technology has emerged rapidly [3]. However, hydrogen is highly flammable with a lower flammability limit of 4% in air. In addition, its colorless, odorless, and buoyant characteristics in air highlight the challenging nature of hydrogen leakage detection for safety [4]. 

Compared with other sensors, such as gas chromatographies, mass spectrometers, optical, or catalytic combustion type sensors, semiconductor-based chemiresistive gas sensors are promising for practical applications due to their low-cost and scalable production methods [5,6,7,8]. The most widely used metal oxide semiconductor(MOS)-based hydrogen sensors are zinc oxide (ZnO), stannic oxide (SnO_2_), tungsten trioxide (WO_3_), titanium dioxide (TiO_2_), niobium pentoxide (Nb_2_O_5_), etc. [9,10]. Among them, ZnO demonstrates many advantages, such as its chemical reactivity towards reducing gas, nontoxicity, low-cost, and suitability for mass production [11,12]. However, most MOS-based hydrogen sensors have shown a limited and slow response and recovery at room temperature. Consequently, MOS-based gas sensors generally need to be heated to a relatively high temperature to increase the response rate. Thus, for the room temperature sensing response, noble nanoparticles are often used [13]. Palladium (Pd) is one of the best catalytic materials for hydrogen because of its chemisorption property with hydrogen at room temperature. The hydrogen-sensing mechanism of the Pd is attributed to the dislocation of the H_2_ molecule to the H atom and the formation of PdHx [2]. The interaction of the hydrogen atoms on the surface of Pd is described by: Pd + H ↔ PdHx. The synthesis of metal nanoparticles is a complex process that requires toxic chemicals and requires multiple steps. Some common approaches to form metal nanoparticles are hydrothermal, electrodeposition, solvothermal, and ablation [14]. Some of the methods mentioned above require separate deposition process steps and leave behind some unwanted impurities such as surfactants. A direct fabrication approach is preferred due to its simplicity. In this study, a thin palladium film on a prefabricated oxide-based sensor using a shadow mask was first sputter-deposited. Then, palladium nanoparticles were obtained by thermally annealing the thin film [15].

Despite the enhancement of the sensor response at room temperature obtained by the noble-metal-decorated, MOS-based H_2_ sensors, selectivity in the presence of interfering gas species is still an issue. Polymer membranes such as poly(methyl methacrylate) (PMMA) and polytetrafluoroethylene (PTFE) have been used to improve the selectivity of hydrogen gas sensors [16,17]. Instead of the physical formation of such membranes, the self-assembly of 1-octadecanethiol, utilizing its long-chain thiols (CnH_2_n_+1_ -SH, n = 18), is proposed in this work. 1-octadecanethiol molecules can spontaneously assemble themselves in stacks of monolayers on the surfaces of solid materials due to chemisorption [18]. This simple process of the formation of the monolayers makes it technologically attractive for surface engineering applications [18]. In addition, it was reported that the use of 1-octadecanethiol could enhance the stability of a semiconductor device in exposure to air due to the sulfur layer [19].

In this paper, we propose a time and cost-effective fabrication technique for a flexible H_2_ sensor via a combination of screen printing, sputtering, and spin-coating. In addition, we explore the use of 1-octadecanethiol as a novel selective hydrophobic membrane for hydrogen sensors. The morphology and the chemical composition of the fabricated device were evaluated using a scanning electron microscope (SEM) and X-ray photoelectron spectroscopy (XPS). The gas-sensing properties, including sensor response, response time, and response to interfering gases, were studied in detail.

## 2. Materials and Methods

### 2.1. Chemicals

Zinc oxide, terpinol, 1-octadecanethiol, and ethanol were purchased from Sigma Aldrich (St. Louis, MO, USA). Ethylene cellulose, silver paste, and kapton^®^ (polyimide) film were obtained from Alfa Assar (Ward Hill, MA, USA), Daejoo Electronic Materials (Gyeonggi-Do, Korea), and CS Hyde Company (lake Villa, IL, USA), respectively. Pd (99.98% purity) used for sputtering was obtained from Tedpella Inc. (Redding, CA, USA). All these chemicals were used without additional treatment or purification.

### 2.2. Prepration of ZnO Paste

First, 2 g of ethylene cellulose was added to 20 mL of ethanol and 78 mL of terpinol was then stirred with a magnetic stirrer for 24 h. Ethylene cellulose was used as a binder to enhance the bonding strength between the sensing material and the substrate. Then ZnO nanoparticle powder was mixed with the prepared solution such that the weight ratio of ZnO to a solution was 60:40. The mixture was stirred further to prepare the final paste. The obtained paste was used for screen-printing without any further treatment.

### 2.3. Device Fabrication

**Figure 1:**
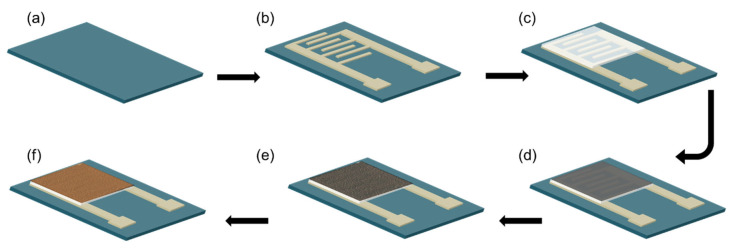
Schematic illustration of the fabrication of a flexible chemical sensor: (**a**) flexible substrate, (**b**) screen printing of silver electrodes, (**c**) screen printing ZnO, (**d**) sputtering palladium, (**e**) annealing step, (**f**) spin-coating polymer layer.

The device fabrication procedure is shown in Figure 1. The mesh screens were designed using AutoCAD and were obtained from NBC Meshtec. Americas Inc. (Batavia, IL, USA). A polyimide film was used as a flexible substrate. First, interdigitated silver electrodes were printed on the polyimide film using commercial screen paste. Then, the ZnO film was printed on top of the planar interdigitated electrodes using the preformulated ZnO paste. After each printing step, samples were dried for 4 h at room temperature.

**Figure 2:**
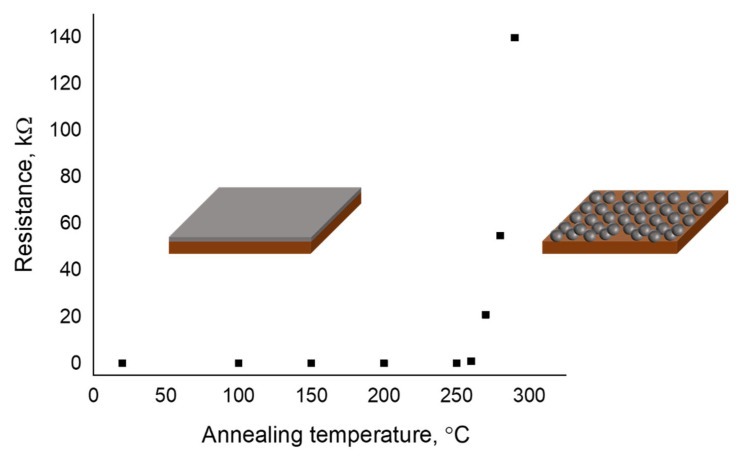
Resistance variance of the thin film with respect to change in the annealing temperature.

Palladium thin films were deposited on top of the ZnO film by DC-magnetron sputtering. The shadow mask was placed close to the substrate during the sputtering step. After this the device was annealed to form palladium nanoparticles. Figure 2 shows the change in the resistance of the thin film with respect to the change in the annealing temperature. The morphology of the palladium nanoparticles was analyzed using SEM. Finally, the 1-octadecanethiol layer was spin-coated as the hydrogen selective layer (Figure 1f).

### 2.4. Materials Characterization

The surface morphologies of the sensors were studied using a Zeiss Ultra 55 scanning electron microscope (SEM) system (Carl Zeiss SMT GmbH, Oberkochen, Germany) operated at 5 keV. X-ray photoelectron spectroscopy (XPS) spectra were collected on ESCALAB™ XI+ X-ray photoelectron spectrometer microprobe (Thermo Scientific, Waltham, MA, USA). A monochromatic, micro-focused Al Kα line was used to analyze the XPS of the sample. The sensor resistance was measured using a Keithley 2401 source meter (Tektronix Inc., Beaverton OR, USA). The sample thickness was measured by spectroscopic ellipsometry (J.A. Woollam Co., Inc., Lincoln, NE, USA).

## 3. Results and Discussions

### 3.1. Pd Nanoparticle Formation

The annealing of the Pd thin film under ambient conditions was studied at different temperatures. The palladium films deposited on the polyimide substrates were annealed at 100 to 300 °C for 30 min. The resistance was measured after annealing. Figure 2 shows the change in the resistance of the thin film with respect to change in the annealing temperature. The resistance of the thin film suddenly increased at an annealing temperature higher than 250 °C. In addition, the sample annealed at higher than 300 °C became electrically resistive. This was expected because of the annealing-induced discontinuity of the film and the formation of nanoparticles. The morphology study using SEM showed the formation of the Pd nanoparticle.

### 3.2. Morphology and Chemical Analysis of Pd/ZnO

**Figure 3:**
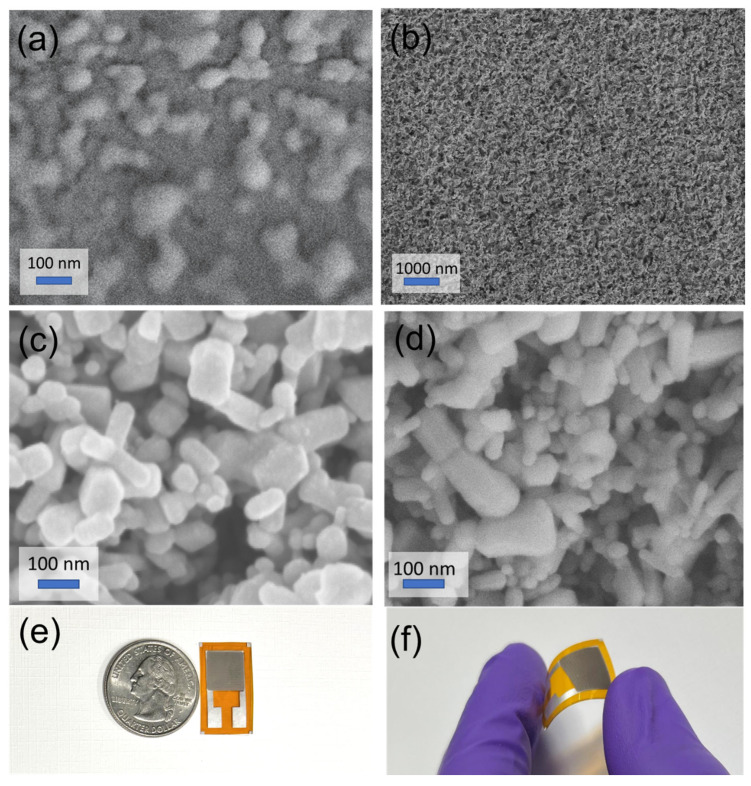
(**a**,**b**) Scanning electron microscope (SEM) image of the palladium film (different magnifications) after annealing to demonstrate the formation of the palladium nanoparticle, (**c**) SEM image of ZnO film, (**d**) SEM image of Pd/ZnO, (**e**) photograph of the fabricated sensor, and (**f**) photograph showing the flexibility of the fabricated sensor.

To analyze the formation of the palladium nanoparticle, first, thin palladium film was deposited on the polyimide substrate. Then samples were annealed at 300 °C, and the SEM image was obtained. Figure 3a,b shows images of the palladium film (different magnifications) after annealing at 300 °C. It is clear from the SEM images that palladium nanoparticles were formed and evenly distributed due to the annealing of the sample. The nanoparticle formation can be attributed to the unequal expansion of the substrate and the palladium film. Figure 3b,c shows the SEM images of the ZnO and Pd/ZnO sensing layer. The SEM image of the ZnO layer shows the average particle size of 108 ± 11 nm. The image (3c) reveals two sets of particles; bigger particles were associated with ZnO, and smaller particles were associated with the palladium nanoparticles. The average size of ZnO nanoparticles and palladium nanoparticles were 115 ± 16 nm and 38 ± 8 nm, respectively. Figure 3e shows a photograph of the fabricated sensor, and Figure 3f shows the flexibility of the fabricated sensor.

**Figure 4:**
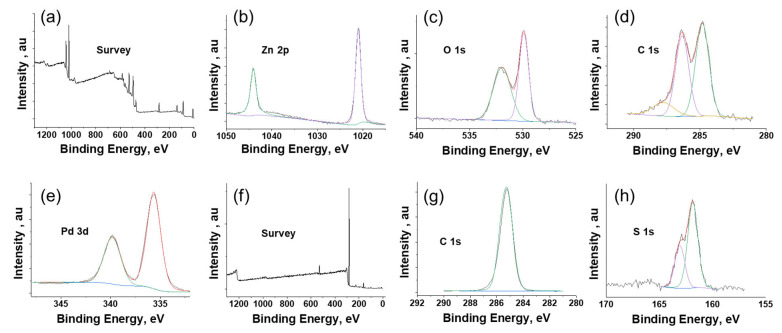
X-ray photoelectron spectroscopy (XPS) analysis of sensor: (**a**) survey peak of ZnO/Pd layer; (**b**) Zn 2p spectra of ZnO/Pd; (**c**) O 1s spectra of ZnO/Pd; (**d**) C 1s spectra of ZnO/Pd; (**e**) P 3d spectra of ZnO/Pd; (**f**) survey peak of membrane; (**g**) C 1s spectra of membrane; and (**h**) S 1s spectra of membrane.

The XPS spectra of Pd/ZnO is presented in Figure 4. The survey peak of Pd/ZnO layer shown in Figure 4a indicates the presence of zinc, oxygen, carbon, and palladium. Figure 4b–e shows the high-resolution XPS spectra of Zn 2p, O 1S, C 1s, and Pd 3d, respectively. The peaks at 1044.1 eV and 1021.1 eV correspond to Zn 2p3/2 and Zn 2p1/2, respectively. The energy-splitting between the Zn 2p1/2 and Zn 2p3/2 was ~23 eV, which agrees with the literature [20]. The two clear, distinct peaks located at 335.2 and 341.1 eV were attributed to the spin-orbit of Pd 2p3/2 and Pd 2p1/2, respectively [21]. The deconvolution of C 1s revealed that there were three chemical states centered at 284.8, 286.4, and 287.9 eV. The peak centered at 284.8 eV is associated with C–O groups in the form of C–OH [20]. The sp^2^ and sp^3^ hybridized carbon peaks are observed at 284.9 and 286.4, respectively. The O 1s core peak (Figure 4c) of ZnO nanoparticles shows one distinct peak at 529.8 eV associated with O^2−^ ions in the Zn–O bonding of the ZnO nanoparticle [20]. The peak around 529.8 eV is associated with C–O groups.

The survey peak of the 1-octadecanethiol polymer membrane shown in Figure 4f indicates the presence of sulfur and carbon. Figure 4g shows the high-resolution spectra of C1s peak. The C 1s single spectrum centered at 285.2 eV is attributed to the alkyl chain of the 1-octadecanethiol molecule; this observation agrees with the literature [22]. Figure 4h shows the high-resolution spectra of S 1s. The deconvolution S 1s peaks at binding energies 163.08 eV and 161.86 eV represent the free sulfur and the interaction of sulfur with carbon, respectively [23]. The results show the successful fabrication of the multilayered sensor.

### 3.3. Polymer Membrane Formation and Characterization

**Figure 5:**
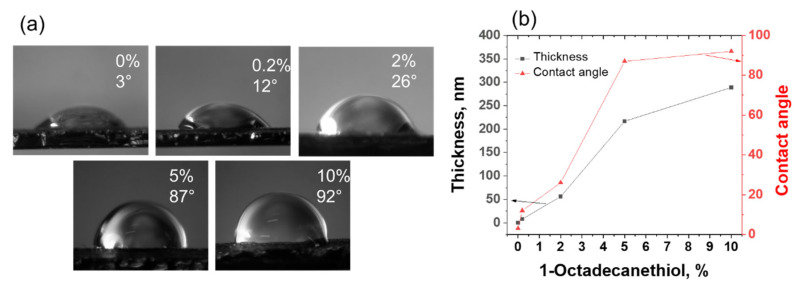
Variation of (**a**) contact angle for water on 1-octadecanethiol film and (**b**) thickness of 1-octadecanethiol film at different concentrations of the coating solution.

Different concentrations of 1-octadecanethiol were dissolved in toluene. A thin film was prepared by spin-coating 1-octadecanethiol on the silicon substrate at a spin rate of 1500 rpm for 30 s. Before contact angle and film thickness measurement, the samples were dried at room temperature for 24 h to remove solvent and relax the polymer chain. The sample thickness was measured by spectroscopic ellipsometry (J.A. Woollam Co., Inc.). To measure contact angle, first, a drop of deionized water (50 µL) was placed on the samples, and images were taken using an optical microscope. Later microscope images were analyzed using ImageJ software to calculate contact angle [24]. Figure 5 shows the variation of the film thickness and contact angles of 1-octadecanethiol with respect to the change in the concentration of the coating solutions. The observed results show that the contact angle was strongly dependent on the film thickness and became saturated with the thicker layer. This shows that the long-range van der Waal’s force with the underlying substrate was dominant for the thinner film.

### 3.4. Gas Sensing Characterization

The fabricated sensors were placed inside the homebuilt chamber to detect hydrogen gas and its interference gases, such as moist air, methane, ethanol, and acetone. The probes were connected to a source meter (Keithley 2401) to measure sensor response. The sensor response was obtained using the following relation.

Response = $\frac{\Delta I}{I_{0}}$ where, ΔI is the change in the current due to exposure of the test gas and $I_{0}$ is the current in the presence of nitrogen.

**Figure 6:**
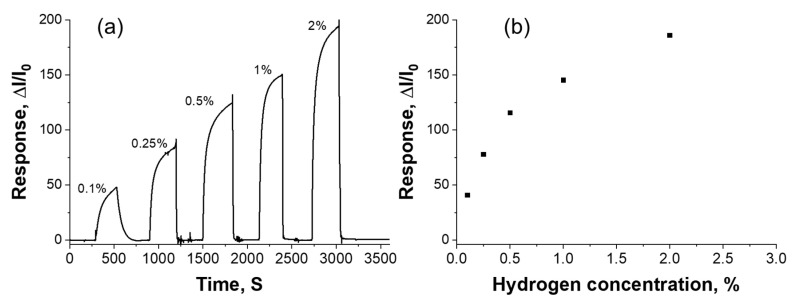
(**a**) The dynamic response of hydrogen ranging from 0.1% to 2%, and (**b**) change in the sensor response with respect to change in the hydrogen concentration.

Figure 6a shows the dynamic response of the sensor to 0.1% to 2% hydrogen concentration at room temperature. A stable and fast response can be observed with the step change in the hydrogen concentration. The response time of the sensor was decreased from 68 s to 43 s with increase in the hydrogen concentration from 0.1% to 2%. The experimental values of the sensor response, with respect to change in the concentration, is shown in Figure 6b. The results show that the sensor response increased from 39.2 to 186 with increase in the hydrogen concentration from 0.1% to 2%.

**Figure 7:**
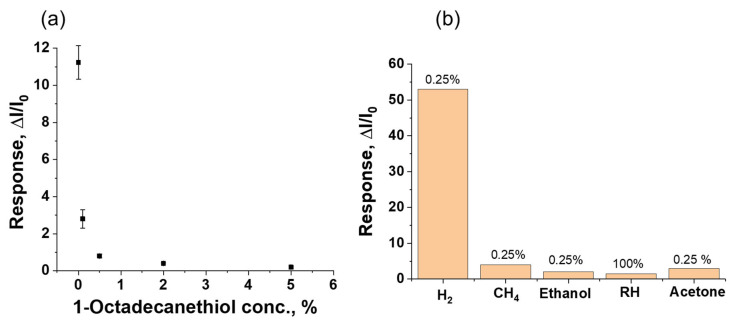
(**a**) The sensor response to moisture (RH = 100%) with change in the concentration of membrane coating solution (thickness), and (**b**) sensor response with other interfering gas.

In this study, a 1-octadecanethiol membrane was used to improve the selectivity of the sensor. The membrane thickness was optimized by comparing the values of the sensor response to 100% humidity before evaluating the H_2_ selective property of the membrane with other interfering gases. Figure 7a shows the response of the sensor towards 100% relative humidity with respect to changes in the concentration of the coating solution. The sensor without membrane showed a strong response to the moisture (RH = 100%), as shown in Figure 7a. In contrast, the sensor response was decreased significantly with an increase in the membrane thickness. Based on the observed result, a 2% coating solution (~60 nm) was used as an optimal membrane thickness. It is clear that the self-assembled 1-octadecanethiol layer can act as a moisture barrier because of low water vapor permeability and its hydrophobicity. To further investigate the sensor selectivity, the sensor was exposed to different interfering gases such as moist air, methane, ethanol, and acetone. The response values obtained for methane, ethanol, and acetone at 0.25% concentrations were 4, 1.5, and 3, respectively. The results show that the sensor had minimum response to the interfering gases. The reason behind the better selectivity of the fabricated sensor is the presence of monolayers of the 1-octadecanethiol polymer membrane.

The detailed comparisons of Pd/ZnO-based hydrogen sensors are summarized in Table 1. The sensor response in the present study was higher than the room temperature Pd/ZnO-based hydrogen sensor. The results show that the surfactant-free and uniform distribution of the palladium nanoparticles played a key role in enhancing the absorption of the hydrogen molecules.

## 4. Conclusions

We have fabricated a room temperature, flexible hydrogen sensor by formulating and printing a ZnO nanoparticle paste, sputtering and annealing a Pd film, and assembling a 1-octadecanethiol membrane layer. The SEM and XPS results showed the successful fabrication of the multilayered construct for the selective hydrogen sensor. The deposition of surfactant-free, uniformly deposited Pd nanoparticles contributed to the enhanced response to hydrogen gas. The fabricated sensor showed a high response of 39.2 to 186 upon exposure to 0.1% to 2% concentration of hydrogen gas at room temperature, respectively. To the best of our knowledge, this work has demonstrated for the first time the use of a 1-octadecanethiol polymer membrane as the selective layer for hydrogen sensors. The 1-octadecanethiol polymer membrane showed good sensor response towards hydrogen over methane, humid air, ethanol, and acetone. In summary, a two-pronged approach employing Pd nanoparticles for high response and a 1-octadecanethiol polymer membrane for a selective membrane was presented as a simplified but effective fabrication method for hydrogen sensors that can operate at room temperature with good characteristics.

## Figures and Tables

**Table 1:** Comparison of the Pd/ZnO based hydrogen sensors.

Materials	Concentration, ppm	Operating Temperature, °C	Response	Response Time, s	Substrate	Ref
Pd/ZnO	1000	RT	0.91	18	Flexible	[25]
Pd/ZnO	360	RT	13	75	Rigid	[26]
Pt/Pd/ZnO	10,000	100	69.8	5	Flexible	[27]
Pd/ZnO	100	RT	1.45	64	Rigid	[28]
Pd/ZnO	1000	RT	39.2	68	Flexible	This work

## References

[1] Li Z., Xu Q. (2017). Metal-nanoparticle-catalyzed hydrogen generation from formic acid. Acc. Chem. Res..

[2] Koo W.-T., Cho H.-J., Kim D.-H., Kim Y.H., Shin H., Penner R.M., Kim I.-D. (2020). Chemiresistive hydrogen sensors: Fundamentals, recent advances, and challenges. ACS Nano.

[3] Ajanovic A., Glatt A., Haas R. (2021). Prospects and impediments for hydrogen fuel cell buses. Energy.

[4] Rigas F., Amyotte P. (2012). Hydrogen Safety.

[5] Nikolic M.V., Milovanovic V., Vasiljevic Z.Z., Stamenkovic Z. (2020). Semiconductor gas sensors: Materials, technology, design, and application. Sensors.

[6] Lin Y., Fan Z. (2020). Compositing strategies to enhance the performance of chemiresistive CO_2_ gas sensors. Mater. Sci. Semicond. Processing.

[7] Lu Y., Hsieh C., Su G. (2019). The role of ALD-ZnO seed layers in the growth of ZnO nanorods for hydrogen sensing. Micromachines.

[8] Bamshad A., Cho H.J. (2021). Laserjet Printed Micro/Nano Sensors and Microfluidic Systems: A Simple and Facile Digital Platform for Inexpensive, Flexible, and Low-Volume Devices. Adv. Mater. Technol..

[9] Luo Y., Zhang C., Zheng B., Geng X., Debliquy M. (2017). Hydrogen sensors based on noble metal doped metal-oxide semiconductor: A review. Int. J. Hydrog. Energy.

[10] Pathak P., Park S., Cho H.J. (2020). A Carbon Nanotube–Metal Oxide Hybrid Material for Visible-Blind Flexible UV-Sensor. Micromachines.

[11] Khayatian A., Kashi M.A., Azimirad R., Safa S. (2014). Enhanced gas-sensing properties of ZnO nanorods encapsulated in an Fe-doped ZnO shell. J. Phys. D Appl. Phys..

[12] Zhu L., Zeng W. (2017). Room-temperature gas sensing of ZnO-based gas sensor: A review. Sens. Actuators A Phys..

[13] Fan F., Zhang J., Li J., Zhang N., Hong R., Deng X., Tang P., Li D. (2017). Hydrogen sensing properties of Pt-Au bimetallic nanoparticles loaded on ZnO nanorods. Sens. Actuators B Chem..

[14] Jamkhande P.G., Ghule N.W., Bamer A.H., Kalaskar M.G. (2019). Metal nanoparticles synthesis: An overview on methods of preparation, advantages and disadvantages, and applications. J. Drug Deliv. Sci. Technol..

[15] Teoh W.Y., Amal R., Mädler L. (2010). Flame spray pyrolysis: An enabling technology for nanoparticles design and fabrication. Nanoscale.

[16] Ngene P., Westerwaal R.J., Sachdeva S., Haije W., de Smet L.C., Dam B. (2014). Polymer-induced surface modifications of Pd-based thin films leading to improved kinetics in hydrogen sensing and energy storage applications. Angew. Chem. Int. Ed..

[17] Hong J., Lee S., Seo J., Pyo S., Kim J., Lee T. (2015). A highly sensitive hydrogen sensor with gas selectivity using a PMMA membrane-coated Pd nanoparticle/single-layer graphene hybrid. ACS Appl. Mater. Interfaces.

[18] Ulman A. (1996). Formation and structure of self-assembled monolayers. Chem. Rev..

[19] Zhou L., Chu X., Chi Y., Yang X. (2019). Property improvement of GaAs surface by 1-octadecanethiol passivation. Crystals.

[20] Guo L., Zhang H., Zhao D., Li B., Zhang Z., Jiang M., Shen D. (2012). High responsivity ZnO nanowires based UV detector fabricated by the dielectrophoresis method. Sens. Actuators B Chem..

[21] Militello M.C., Simko S.J. (1994). Elemental palladium by XPS. Surf. Sci. Spectra.

[22] Cavalleri O., Oliveri L., Dacca A., Parodi R., Rolandi R. (2001). XPS measurements on L-cysteine and 1-octadecanethiol self-assembled films: A comparative study. Appl. Surf. Sci..

[23] Ishida T., Hara M., Kojima I., Tsuneda S., Nishida N., Sasabe H., Knoll W. (1998). High resolution X-ray photoelectron spectroscopy measurements of octadecanethiol self-assembled monolayers on Au (111). Langmuir.

[24] Lamour G., Hamraoui A., Buvailo A., Xing Y., Keuleyan S., Prakash V., Eftekhari-Bafrooei A., Borguet E. (2010). Contact angle measurements using a simplified experimental setup. J. Chem. Educ..

[25] Rashid T.-R., Phan D.-T., Chung G.-S. (2013). A flexible hydrogen sensor based on Pd nanoparticles decorated ZnO nanorods grown on polyimide tape. Sens. Actuators B Chem..

[26] Kashif M., Ali M., Ali S.M.U., Hashim U. (2013). Sol–gel synthesis of Pd doped ZnO nanorods for room temperature hydrogen sensing applications. Ceram. Int..

[27] Hassan K., Uddin A.I., Ullah F., Kim Y.S., Chung G.-S. (2016). Platinum/palladium bimetallic ultra-thin film decorated on a one-dimensional ZnO nanorods array for use as fast response flexible hydrogen sensor. Mater. Lett..

[28] Lupan O., Postica V., Labat F., Ciofini I., Pauporte T., Adelung R. (2018). Ultra-sensitive and selective hydrogen nanosensor with fast response at room temperature based on a single Pd/ZnO nanowire. Sens. Actuators B Chem..

